# Whole-Genome Sequences of *Bacillus* and *Paenibacillus* sp. Strains Isolated from Honey in Japan

**DOI:** 10.1128/mra.00842-22

**Published:** 2022-10-27

**Authors:** Kayo Okumura, Mariko Okamoto, Daisuke Takamatsu

**Affiliations:** a Department of Veterinary Medicine, Obihiro University of Agriculture and Veterinary Medicine, Obihiro, Hokkaido, Japan; b Division of Infectious Animal Disease Research, National Institute of Animal Health, National Agriculture and Food Research Organization, Tsukuba, Ibaraki, Japan; c The United Graduate School of Veterinary Sciences, Gifu University, Gifu, Gifu, Japan; University of Rochester School of Medicine and Dentistry

## Abstract

Knowledge about bacterial species in bee environments is essential for maintaining healthy honeybee colonies. Therefore, we performed whole-genome sequence analysis on bacteria isolated from honey harvested in Japan. This study reports the genomic sequences of the five bacterial strains identified.

## ANNOUNCEMENT

Although honeybees are essential livestock for pollination and providing honey, research on infectious diseases affecting honeybees is not comprehensive. Previously, we isolated bacteria from honey in Japan (1) and performed whole-genome shotgun sequencing to investigate honeybee pathogens. However, obtaining complete genomic sequences is key to comprehensively understanding the toxicity and genetic profiles of the bacteria. In this study, we performed complete genomic analysis of five representative strains. Here, we report the complete genomic data for Bacillus paralicheniformis and *Paenibacillus* spp. found in honey.

Bacteria isolated from honey and stored as glycerol stocks were subsequently recovered and their genomic DNA purified (1). Bacteria were cultured on Columbia agar with 5% defibrinated sheep blood, harvested, and suspended in 550 μL Tris-EDTA buffer. The cells were treated with approximately 10 mg/mL lysozyme and 50 U/mL mutanolysin (Sigma-Aldrich, USA) at 37°C and lysed with 1% sodium dodecyl-sulfate. The lysates were mixed with once with phenol, thrice with phenol-chloroform-isoamyl alcohol (PCI), and once with chloroform to extract DNA and remove the proteins. After ethanol precipitation, 100 μg/mL RNase (Nippon Gene, Japan) treatment, PCI and chloroform extraction, ethanol precipitation, and a 70% ethanol rinse, the purified DNA was suspended in 10 mM Tris-HCl (pH 8.5) and stored at −20°C.

Whole-genome sequencing was performed using the PacBio RS II platform combined with a SMRT cell 8Pac ver. 3 and a DNA polymerase binding kit P6 (Pacific Biosciences, USA). For Illumina sequencing, the NovaSeq 6000 (Illumina, USA) system was used with 150-bp paired-end read settings. Samples were prepared using the TruSeq DNA PCR free kit (Illumina, USA) per the manufacturer’s instructions. Read quality control, raw read filtering, and *de novo* sequence assembly were performed using HGAP ver. 3 software (Pacific Biosciences) (2) and polished using Quiver (2) with default settings. The assembly accuracy was validated by mapping Illumina reads obtained in a previous study (1) using Pilon ver. 1.21 (3) with default settings, with 100.00% coverage (except for Paenibacillus azoreducens J34TS1; 99.97%). After assembly, a self-dot plot was created using UGENE ver. 35.0 (http://ugene.net) to verify the plasmid and chromosome circularization by checking the contig end overlap and the 5′ and 3′ end connections. Analysis was performed with BUSCO ver. 3.0 software (4), using the bacteria_odb9 database to validate the completeness of genome assembly; for all genomes, quality scores of >98.7% were obtained. The general features of each genome are listed in Table 1.

**TABLE 1:** General features of the bacterial genomes presented in this study^*a*^

Species	Isolate	Total no. of subreads	*N*_50_ (bp)	Total no. of subread bases	Size (bp)	GC content (%)	Coverage (×)	BioSample accession no.	SRA accession no.
Bacillus paralicheniformis	J25TS1	78,163 (P), 37,219,662 (I)	16,453	708,855,100 (P), 5,620,168,962 (I)	4,394,709	45.9	129 (P), 1,152 (I)	SAMD00425495	DRX320385 (P), DRX321129 (I)
	J36TS2	74,813 (P), 22,269,482 (I)	15,585	651,721,606 (P), 3,362,691,782 (I)	4,404,119	45.9	116 (P), 678 (I)	SAMD00425496	DRX320386 (P), DRX321130 (I)
	J36TS2 plasmid^*b*^				200,793	36.5	159 (P)		
	J41TS8	90,978 (P), 22,392,292 (I)	15,975	914,167,224 (P), 3,381,236,092 (I)	4,501,066	45.8	171 (P), 699 (I)	SAMD00425497	DRX320387 (P), DRX321131 (I)
Paenibacillus azoreducens	J34TS1	96,372 (P), 36,125,506 (I)	13,745	840,311,025 (P), 5,454,951,406 (I)	7,316,152	48.0	96 (P), 676 (I)	SAMD00425498	DRX320388 (P), DRX321132 (I)
Paenibacillus dendritiformis	J27TS7	130,145 (P), 32,933,880 (I)	15,307	1,359,398,548 (P), 4,973,015,880 (I)	6,547,390	54.7	124 (P), 690 (I)	SAMD00425499	DRX320389 (P), DRX321133 (I)

a The total number of subreads and subread bases and the coverage depth values were obtained by PacBio (P) and Illumina (I) sequencing. The *N*_50_, size, and GC content values were obtained by PacBio sequencing.

b *B. paralicheniformis* J36TS2 has a single plasmid, pBP1.

Few genomes of P. azoreducens and Paenibacillus dendritiformis have been registered. Although NCBI contains genomic information on approximately 100 *B*. *paralicheniformis* strains, complete genome sequences are absent for honey-derived strains. The data obtained in this study are valuable for understanding bacteria in bee environments to promote future research.

### Data availability.

The whole-genome sequences have been deposited at DDBJ/GenBank under BioProject accession number PRJDB12645 and BioSample accession numbers SAMD00425495 to SAMD00425499 (Table 1). The raw sequence reads have been deposited in the DDBJ DRA/NCBI SRA, and the accession numbers are listed in Table 1.
